# Evaluating human papillomavirus vaccination programs in Canada: should provincial healthcare pay for voluntary adult vaccination?

**DOI:** 10.1186/1471-2458-8-114

**Published:** 2008-04-10

**Authors:** Marco Llamazares, Robert J Smith?

**Affiliations:** 1 Department of Mathematics, The University of Ottawa, Ottawa, Canada; 2 Faculty of Medicine, The University of Ottawa, Ottawa, Canada

## Abstract

**Background:**

Recently, provincial health programs in Canada and elsewhere have begun rolling out vaccination against human papillomavirus for girls aged 9–13. While vaccination is voluntary, the cost of vaccination is waived, to encourage parents to have their daughters vaccinated. Adult women who are eligible for the vaccine may still receive it, but at a cost of approximately CAN$400. Given the high efficacy and immunogenicity of the vaccine, the possibility of eradicating targeted types of the virus may be feasible, assuming the vaccination programs are undertaken strategically.

**Methods:**

We develop a mathematical model to describe the epidemiology of vaccination against human papillomavirus, accounting for a widespread childhood vaccination program that may be supplemented by voluntary adult vaccination. A stability analysis is performed to determine the stability of the disease-free equilibrium. The critical vaccine efficacy and immunogenicity thresholds are derived, and the minimum level of adult vaccination required for eradication of targeted types is determined.

**Results:**

We demonstrate that eradication of targeted types is indeed feasible, although the burden of coverage for a childhood-only vaccination program may be high. However, if a small, but non-negligible, proportion of eligible adults can be vaccinated, then the possibility of eradication of targeted types becomes much more favourable. We provide a threshold for eradication in general communities and illustrate the results with numerical simulations. We also investigate the effects of suboptimal efficacy and immunogenicity and show that there is a critical efficacy below which eradication of targeted types is not possible. If eradication is possible, then there is a critical immunogenicity such that even 100% childhood vaccination will not eradicate the targeted types of the virus and must be supplemented with voluntary adult vaccination. However, the level of adult vaccination coverage required is modest and may be achieved simply by removing the cost burden to vaccination.

**Conclusion:**

We recommend that provincial healthcare programs should pay for voluntary adult vaccination for women aged 14–26. However, it should be noted that our model results are preliminary, in that we have made a number of simplifying assumptions, including a lack of age-dependency in sexual partner rates, a lack of sexual activity outside of the vaccine age-range among females and a uniform age of sexual debut; thus, further work is desired to enhance the external generalisability of our results.

## Background

Human papillomavirus (HPV) produces epithelial tumors of the skin and mucous membranes [1]. There are over 100 types, many of which are relatively benign. However, some types have emerged as high risk because they produce lesions that may lead to carcinomas [2]. Resulting disease includes genital warts, respiratory papillomatosis, and cancer of the cervix, vulva, vagina, anus and the penis [2], as well as cancers of the head and neck [3]. Prevalence of HPV in Canada has been estimated at 24% in female university students [4]. In Ontario, 500 women are diagnosed with cervical cancer annually, leading to 140 deaths [5]. Between 30 and 40 types are transmitted through sexual contact. Without condoms, risk of transmission, given contact with an infected partner, is close to 90%; this risk is still high (40%) when condoms are used [6]. No antivirals have been developed for HPV and detection has largely relied on the recommended yearly pap smear, which locates cellular abnormalities that indicate that HPV may be present [7].

Cervical cancer is the second most common cause of death in women (after breast cancer) and it accounts for 10% of all cancers in women [8]. Progression to malignancy after acquisition of HPV usually takes at least 10 years [8]. Types 16 and 18 account for approximately 70% of these cervical cancers [8-10]. Merck and GlaxoSmithKline (GSK) have developed commercial vaccines which target types 16 and 18 [8]. Merck's vaccine also protects against types 6 and 11, which are responsible for 90% of external genital warts [8]. The combination of a successful vaccine and vaccination strategy, in combination with the yearly pap smear, seems to be the best approach towards preventing cervical cancer. The vaccine has been approved for women aged 9–26 [11]. Current studies are showing 90–100% efficacy as well as over 98% immunogenicity rates [8,12] for existing vaccines, with no loss of immunity for at least 5 years [12]. Current vaccination programs against HPV [13-19] consider two distinct groups: girls who have not yet begun to be sexually active and women under 26 who are sexually active.

Vaccination programs in Canada have begun in several provinces (Ontario, Nova Scotia, Prince Edward Island) in Fall 2007, targeting school children aged 9–13 [20]. The vaccine is covered by Canadian healthcare for girls in this age group, but is voluntary [5]. Sociological research suggests that about 77% of parents would immunize their children with the HPV vaccine [21]. Vaccination of women aged 14–26 is available, but not covered by provincial healthcare, costing approximately CAN$400 [5] for a full course (three doses) [8]. State-funded voluntary HPV vaccination for adults has been underway in other countries, such as France and Australia, since early 2007 [22]. Possible limitations of a vaccination program include: i) the vaccine may only be delivered to a proportion *p *of the population, ii) the vaccine may only confer immunogenicity in a proportion *ε *of the vaccinated population (ie the vaccine may not always take when administered to the patient), iii) the vaccine may have incomplete efficacy *ψ *(ie the vaccine doesn't always protect against infection during sexual intercourse), iv) the vaccine may wane over time and v) the vaccine does not target all HPV types.

In this paper, we develop a mathematical model to evaluate the effectiveness of supplementing a childhood vaccination program with voluntary adult vaccination. We address the following research questions: 1. Can a childhood-only vaccination program eradicate targeted types of HPV? 2. Should an adult vaccination program supplement childhood vaccination? 3. Is eradication of targeted types possible for vaccines with suboptimal efficacy or immunogenicity?

## Results and Discussion

**Figure 1:**
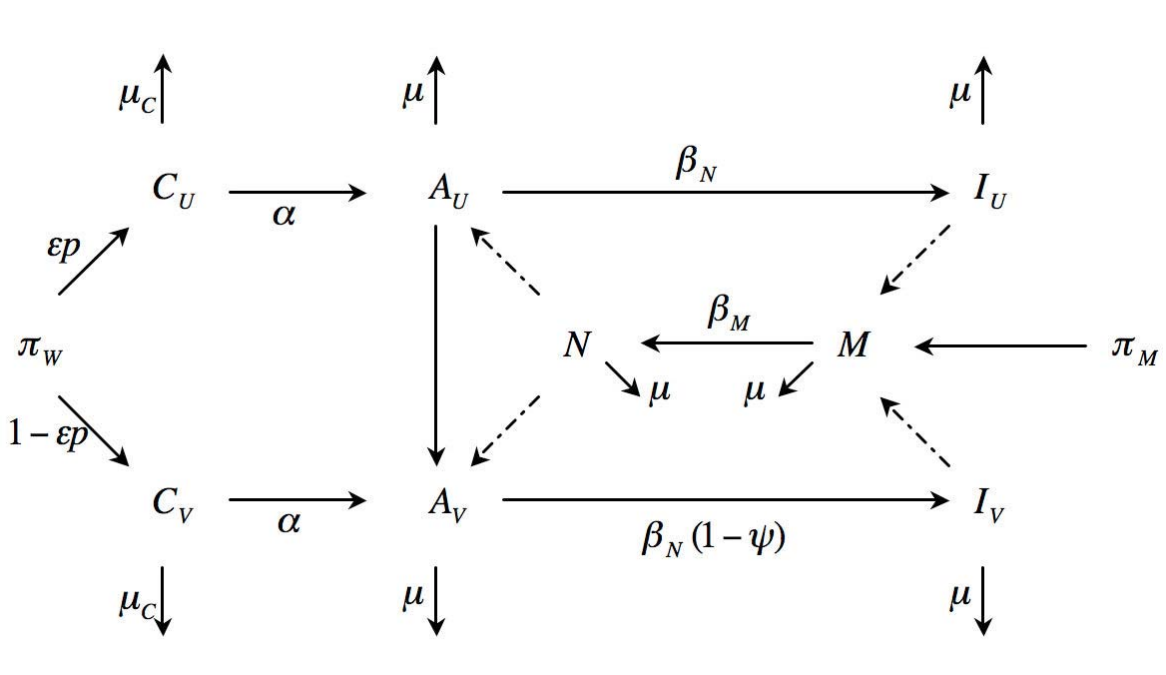
**The model**. Schematic representation of the model representing flow of individuals from pre- to post-vaccination. School-aged girls may be vaccinated (*C*_*V*_) or unvaccinated (*C*_*U*_), depending on the proportion vaccinated (*p*) and the immunogenicity of the vaccine (*ε*). Children progress to sexually active adults at rate *α*. Unvaccinated adult women (*A*_*U*_) can either be vaccinated (*f*) or become infected (*I*_*U*_), with probability *β*_*N*_, when they meet an infected man *N *. Vaccinated adult women (*A*_*V*_) can also become infected (*I*_*V*_), but with reduced transmissibility, due to the efficacy of the vaccine *ψ*. Unvaccinated men (*M*) become infected upon contact with infected women, with transmission probability *β*_*M*_. The mortality rate of children is *μ*_*C *_and the leaving rate of adults is *μ*.

The mathematical model considers female children who may be vaccinated or unvaccinated. Female children grow into sexually active adult women, whereupon unvaccinated adult women may become vaccinated, or they may be infected with HPV. Vaccinated women may still be infected, although they will have a lower probability of infection, due to the vaccine. Men are also included in the model and may be susceptible or infected, but cannot be vaccinated. See Methods for details. The model is illustrated in Figure 1.

There is a threshold of eradication of targeted types given by

$$\varepsilon p=\frac{1}{\psi \mu }\left[\mu +f(\bar{\varepsilon }\bar{p})(1-\psi )-\frac{\mu^{4}(\mu +f(\bar{\varepsilon }\bar{p}))(\alpha +\mu_{C})}{\beta_{M}\beta_{N}\pi_{M}\pi_{W}\alpha }\right],$$

with parameters as given in Table 1. See Methods for mathematical details. Note that, if the vaccination is not at all efficacious (*ψ *= 0), or if the vaccine confers no immunogenicity to children (*ε *= 0), then it is not possible to eradicate targeted types of the disease, regardless of how many children or adults are vaccinated.

**Table 1:** List of symbols

Symbol	Definition	Sample value used	Parameter range
*C*_*U*_	Unvaccinated female children	(state variable)	
*A*_*U*_	Unvaccinated adult women	(state variable)	
*C*_*V*_	Vaccinated female children	(state variable)	
*A*_*V*_	Vaccinated adult women	(state variable)	
*I*_*U*_	Uninfected adult women	(state variable)	
*I*_*V*_	Infected adult women	(state variable)	
*M*	Uninfected men	(state variable)	
*N*	Infected men	(state variable)	
*π*_*W*_	Rate of appearance of new females	50 per year	0–100
*π*_*M*_	Rate of appearance of new males	50 per year	0–100
*ε*	Vaccine immunogenicity in female children	98%	75–98%
$\bar{\varepsilon }$	Vaccine immunogenicity in adult women	98%	75–98%
*p*	Proportion of female children vaccinated	77%	0–100%
$\bar{p}$	Proportion of adult women vaccinated	40%	0–100%
*μ*_*C*_	Mortality rate of children	1/70 years ^-1^	1/140–1/13
*μ*	Leaving rate of adults	1/10 years^-1^	1/12–1
*α*	Rate of progression of female children to sexually activity	$\frac{1}{13-1/\mu }{\text{years}}^{-1}$	1/12–1
*c*	Attentuation constant	0.15 years^-1^	0–0.3
*γ*	Maximal possible rate of adult vaccination	0.1	0–0.2
$f(\bar{\varepsilon }\bar{p})$	Rate at which unvaccinated adult women are vaccinated	$\frac{c\bar{\varepsilon }\bar{p}}{1-\bar{\varepsilon }\bar{p}+\gamma }$	
*β*_*N*_	Probability of infection of a woman by an infected man	0.00056	0–0.00112
*β*_*M*_	Probability of infection of a man by an infected woman	0.0003	0–0.0006
*ψ*	Vaccine efficacy	95%	85–95%

Sample parameters were chosen so that $\frac{1}{\mu }+\frac{1}{\alpha }=13$ (ie the total time between vaccination and leaving was always 13 years), *γc*^-1 ^= 16.67 (ie the optimum age of vaccination for a perfect vaccine with 100% coverage was 16 years 9 months), *β*_*N *_> *β*_*M *_(ie the transmission probability from men to women was higher than from women to men), the total prevalence without vaccination was 24% and so that the proportion of adult-only vaccination was equal to the proportion of childhood-only vaccination required to eradicate targeted types.

We chose sample parameters to illustrate this threshold, using the following key assumptions: females enter the model as children at age 13, the mean rate of progression to sexually active adults is 3 years, women are in the sexually active pool for 10 years (ie until age 26, after which they cannot be vaccinated, so they are no longer under consideration), male partners are also in the pool for 10 years, the vaccine may not confer 100% protection and the probability of transmission from men to women is higher than the probability of transmission from women to men. Although women can still transmit the virus after age 26, we make the simplifying assumption that they will only infect male partners who do not continue to find new, younger female partners. Thus, while 50 year old men may have sexual relationships with 20 year old women (and may have multiple partners among this cohort), we assume that, after age 60, they do not begin relationships with a new cohort of 20 year old women. Parameters were also chosen so that the prevalence of HPV in the sexually active cohort was 24%, in line with Canadian estimates [4]. The maximum possible vaccination rate was set so that, if 100% of sexually active adults were vaccinated with a perfect vaccine, then the average woman would be vaccinated by 16 years 9 months. See the Table for parameters used and sample values.

**Figure 2:**
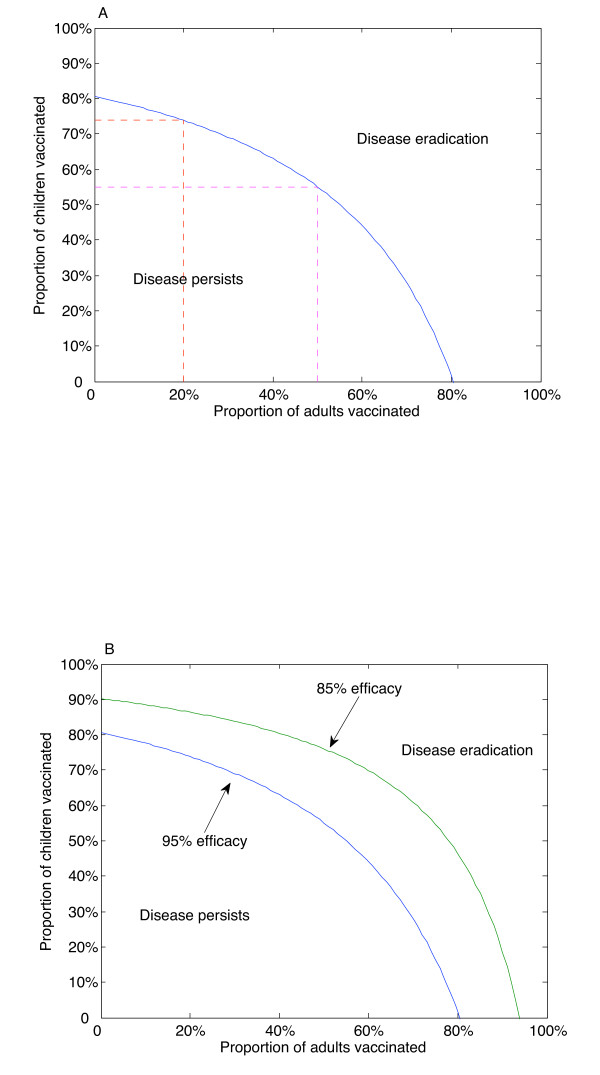
**Thresholds of eradication**. Thresholds of eradication of targeted types for vaccination coverage levels in children versus adults. A. Threshold curve assuming 95% efficacy. Parameters are given in the Table. If no adult vaccination is undertaken, then a childhood vaccination program must cover 81% of the school-aged population for eradication of targeted types. If 20% of adults are vaccinated, then the burden of childhood vaccination reduces to 74%. However, if 50% of adults are vaccinated, then eradication of targeted types can be achieved with only 55% of children vaccinated. B. If the efficacy of the vaccine is reduced to 85%, the eradication threshold is increased, assuming all other parameters remain the same.

A childhood-only vaccination program can eradicate targeted types of the disease, assuming 95% efficacy of the vaccine, if 81% of children are vaccinated (Figure 2A). However, if adult vaccination is included, the burden of childhood vaccination falls; if 20% of adults are vaccinated, then eradication of targeted types could be achieved with 74% childhood vaccination coverage. However, if 50% of adults are vaccinated, then eradication of targeted types could be achieved with only 55% childhood vaccination coverage. If no children were to be vaccinated, then eradication could still be achieved, assuming 81% of adults were vaccinated. As efficacy is reduced, these thresholds increase (Figure 2B). In particular, for our parameter set, the burden of childhood vaccination coverage without adult vaccination rises to 90% when the efficacy is reduced to 85%.

**Figure 3:**
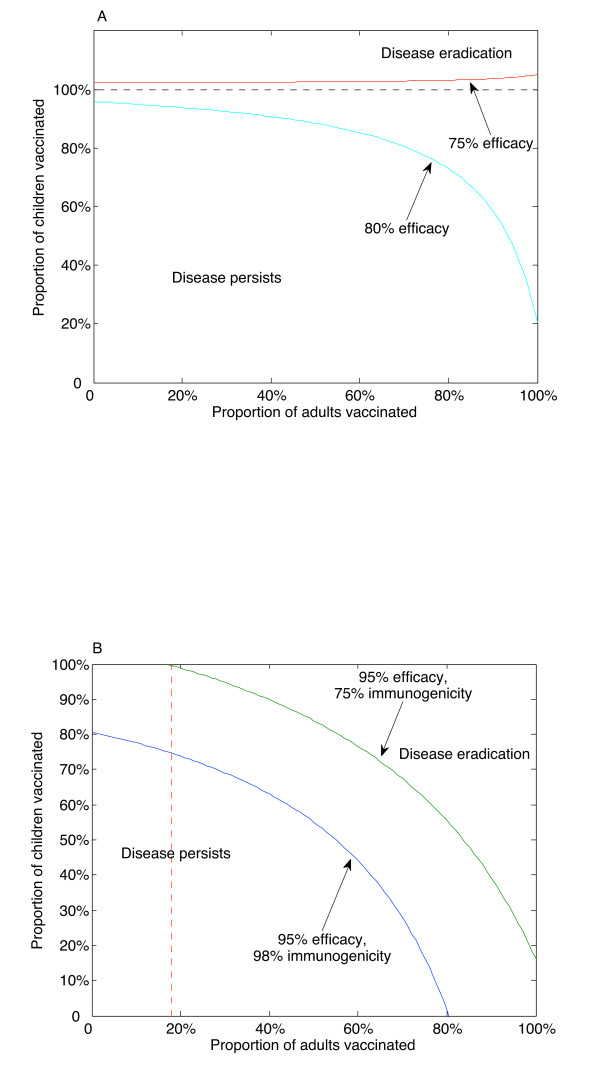
**Suboptimal efficacy and immunogenicity**. Suboptimal values of the efficacy or the immunogenicity lead to qualitative changes in the outcome. A. There is a critical vaccine efficacy (77% in this example), such that no amount of vaccination can eradicate targeted types of the disease if the efficacy falls below this critical value. In this case, eradication of targeted types could only occur if more than 100% of children were vaccinated, which is not possible. B. If the vaccine efficacy allows for eradication of targeted types, there is a critical vaccine immunogenicity (80% in this example), such that even 100% of childhood vaccination will not eradicate targeted types of the disease. In this case, there is a minimum level of adult vaccination coverage that is required for eradication of targeted types (18% if the immunogenicity falls to 75%), even if 100% childhood coverage levels can be achieved. Adult immunogenicity was assumed to be equal to childhood immunogenicity. All other parameters as in the Table.

If the efficacy is reduced further, then eradication of targeted types is no longer possible (Figure 3A).

There is a critical vaccine efficacy ψ*, satisfying

$$\psi^{*}=1-\frac{\mu^{4}(\mu +f)(\alpha +\mu_{C})}{\beta_{M}\beta_{N}\pi_{M}\pi_{W}\alpha },$$

such that, if *ψ *<ψ*, then eradication of targeted types is not possible. See Methods for details. Thus, even if the vaccine mounts an immune response 100% of the time and we can vaccinate 100% of the population, if the efficacy is below this threshold (77% in our example), then the disease will persist. If the immunogenicity is reduced, then eradication of targeted types is not possible with a childhood-only vaccination program (Figure 3B). There is a critical immunogenicity value, ε*, satisfying

$$\varepsilon^{*}=\frac{1}{\psi }\left[1-\frac{\mu^{4}(\alpha +\mu_{C})}{\beta_{N}\beta_{M}\pi_{M}\pi_{W}\alpha }\right],$$

such that, if *ε *<ε*, then even 100% childhood vaccination coverage will not eradicate targeted types of the disease. In this case (assuming *ψ *> ψ*), there is a critical proportion of adults who must be vaccinated. In our example, in the unrealistic case that 100% of children are vaccinated, we would still require 18% of adults to be vaccinated to achieve eradication of targeted types if the immunogenicity is reduced to 75% (Figure 3B).

**Figure 4:**
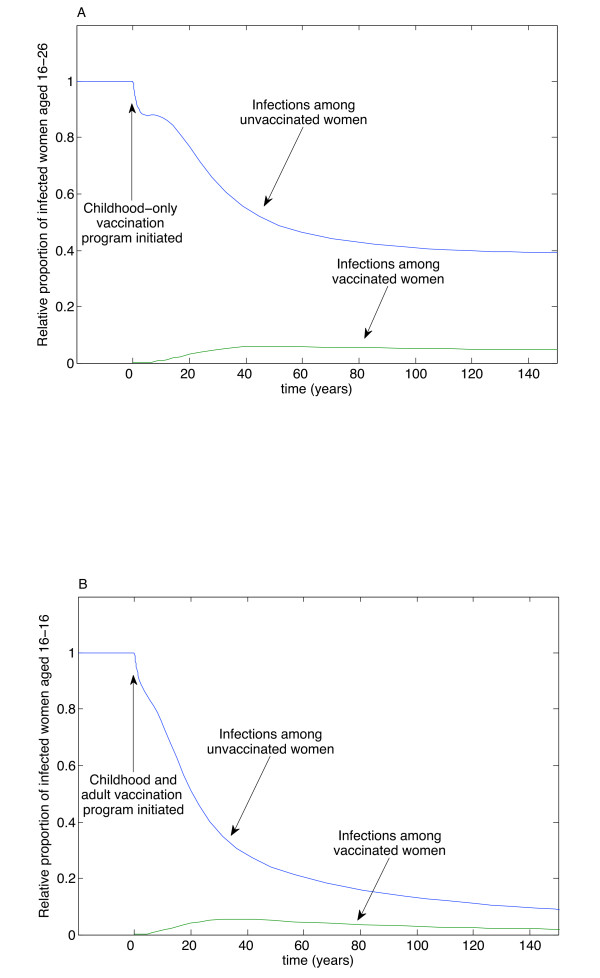
**Timecourse of eradication**. Time course dynamics among a community where initial conditions are taken to be the equilibrium values of an existing infection, for a vaccine with 95% efficacy and 98% immunogenicity. A. If 65% of children are vaccinated, but no adult vaccination occurs, the prevalence in the sexually active cohort is reduced to approximately half of the previous disease burden after 150 years and remains so thereafter. B. If 65% of children and 40% of adults are vaccinated, the number of infections in the sexually active cohort continues to decline, eventually approaching zero. All other parameters as in Figure 4A.

The time course for eradication of targeted types, as with eradicating any disease, is a long one. We illustrate the temporal dynamics in Figure 4. Here, we model a community where the initial condition is the equilibrium infection among women in the sexually active cohort we are investigating. (We stress that this number will be much smaller than the total disease burden in this community.) To demonstrate the importance of adult vaccination, we model two scenarios: in the first, 65% of children are vaccinated, using a vaccine with 95% efficacy and 98% immunogenicity, but no adults are vaccinated. In this case, the number of infections is reduced by about half (Figure 4A), but is not eradicated after more than a century. However, if the same vaccination program is administered, but, additionally, 40% of adults are vaccinated, then the number of infections approaches zero (Figure 4B).

**Figure 5:**
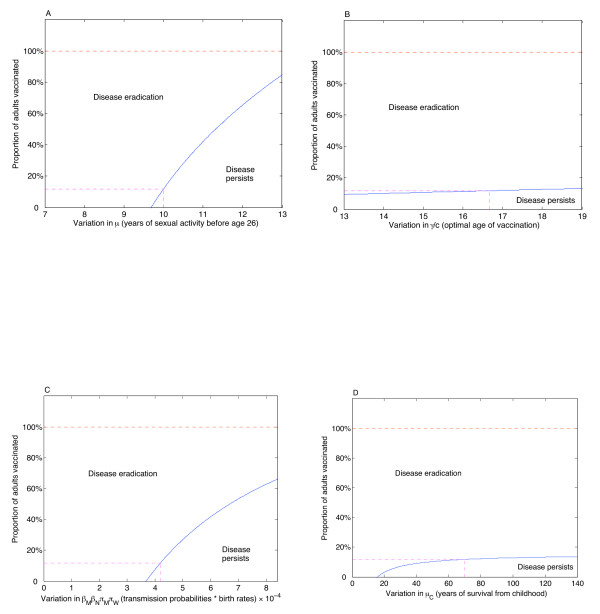
**Dependence on parameter variation**. Sensitivity of eradication threshold on parameter variation, assuming 77% childhood vaccination. A. If the average length of sexual activity before age 26 is 9 and a half years or less, then no adults need be vaccinated. If this time increases, then the threshold also increases, but still remains below 100%, even if children begin sexual activity at age 13. B. Variation as the optimal age of vaccination varies. This rate measures the age at which adult women are vaccinated, assuming 100% of adults are vaccinated with a perfect vaccine. In this case, there is little variation in the output. C. Variation in the transmission probabilities and birth rates. These parameters are always linked, so we examine them as a single unit. Even if these parameters collectively double, eradication could still be achieved with 65% adult vaccination. D. Variation in the childhood mortality rate. The output is relatively steady, unless mortality is very high. In this (unlikely) scenario, life expectancy of 30 years (17 years after vaccination at age 13) or less would clearly obviate the need for an adult vaccination program.

The dependence of the results on parameter variation was also determined. While equation (3) is independent of the sample parameters we chose, and thus is effectively a robust sensitivity analysis unto itself, we illustrate the variation among likely parameter ranges, as given in the Table. The results are illustrated in Figure 5. We use the output variable as the proportion of adults who must be vaccinated in order to achieve eradication of targeted types, assuming that 77% of children are vaccinated [21]. If women spend less time sexually active, then the threshold may be reduced to zero (Figure 5A). Conversely, if women spend more time sexually active, then the threshold may rise considerably, although it will not exceed 100%, even if girls become sexually active as early as 13, which we argue is unlikely for the Canadian average. If the optimal age of vaccination with perfect coverage, *γ*/*c*, increases, then the threshold increases slightly (Figure 5B), but there is little variation even if a perfect vaccine can reach all "adult" women by age 13. Since parameters *β*_*N*_, *β*_*M*_, *π*_*M *_and *π*_*W *_always appear together in (3), we can consider them as a single unit. Even if the transmission probabilities or birth rates collectively double, then the threshold will only increase to 65% required adult vaccination (Figure 5C). Finally, there is little dependence on childhood mortality, as expected (Figure 5D); if life expectancy is significantly shorter than 70 years, then the threshold will be reduced, but we do not expect much variation in this parameter, given overall Canadian life expectancies.

## Conclusion

Despite human papillomavirus being one of the most prevalent sexually transmitted infectious diseases [23], eradication of targeted types is not only possible, but is feasible, under existing vaccination programs, if adults are included. While eradication of targeted types using a childhood-only vaccination program is possible for vaccines with currently estimated efficacy (90–99%) and immunogenicity (98%), it nevertheless poses an extremely high burden of coverage that must be met if only children are to be vaccinated. Crucially, sociological surveys of parents indicated that only 77% were willing to immunise their children with the vaccine [21]. Even in the best-case scenario of a vaccine with 95% efficacy and 98% immunogenicity, our results demonstrate that this proportion of children vaccinated is insufficient for eradication unless a small, but nontrivial, proportion of adults (approximately 16%) is also vaccinated (Figure 2A).

While vaccinating children is desirable, given that the majority of children can be located via the school system, our model demonstrates that any such vaccination program should be supplemented by an adult vaccination program. A widespread adult vaccination program may be unfeasible; however, our results indicate that eradication of targeted types is possible if the proportion of vaccinated children is large, while the proportion of vaccinated adults is modest. Since we do not need to locate all, or even most, sexually active young adults, the required coverage may be achieved merely by not posing obstacles to voluntary adult vaccination. Consequently, we recommend that adult vaccination in Canada be paid for by provincial healthcare, just as both childhood and adult vaccination of Hepatitis B currently is.

By determining an eradication threshold (see Methods), our model can also be used to examine scenarios outside the range of currently available data. Specifically, we investigated the effects that suboptimal efficacy or immunogenicity of the vaccine may have. Although these values have been high in the literature, such data have been taken from clinical trials, which have a relatively small sample size. With a vaccination program targeting a significant number of children, we allowed for the possibility that these effects may not be as high in the broader population. We demonstrated that there is a critical vaccine efficacy, below which eradication of targeted types is not possible, regardless of how many people are vaccinated. We also showed that there is a critical vaccine immunogenicity, below which even 100% childhood vaccination coverage will not lead to eradication of targeted types. Thus, a small but nonzero proportion of adult vaccination coverage is crucial for eradication of targeted types in this case.

A number of cohort, dynamic and population models have been developed in the literature, most of which show that vaccinating women can be cost-effective [24]. Most mathematical models have consistently predicted a useful role for vaccination, although assumptions about naturally acquired immunity and heterogeneity in risk behaviours have varied [26]. One such model, developed before the vaccine was made available, predicted a 30% reduction in epidemic prevalence if females were vaccinated [25]. However, this model assumed the vaccine would only be 75% effective, in line with our conclusions. The annual proportion of cervical cancer cases prevented was shown to be much higher when early adolescents were targeted, in a modelling study based on vaccination in Finland [27]. The authors recommended the implementation of catch-up vaccination at the start of a vaccination programme (ie vaccinating outside the target age group) in order to increase the speed with which a decrease in HPV and cervical cancer incidence is observed. Vaccinating girls and women in a catch-up program in the United States was estimated to be cost-effective, relative to other commonly accepted healthcare programs [28].

Our model has several limitations, which should be noted. First, we assume that the vaccine does not wane. Although this is consistent with current understandings of the vaccine, we only have five years of data to rely on. We assume that men who have sexual relations with women in the sexually active cohort (ie women who are eligible for adult vaccination) do not continue to find new partners in this age group as time goes on. Thus, while 50 year old men may sometimes have sexual relations with (multiple) 25 year old women, we assume that those same men stop having sexual relations with new 25 year olds after a certain period of time. In this way, the sexually active cohorts of men and women are linked only for the time (approximately 10 years) that adult women are sexually active and eligible for vaccination. We also assume that the vaccine plays no role in modifying the nature of the disease for infected vaccinated women; consequently, we assume that men infected by vaccinated women have the same probability of infection as men infected by unvaccinated women. However, we argue that if this were not the case, then the overall burden of infection would only be lowered, so our results are conservative.

It should also be noted that achieving actual eradication of targeted strains may be difficult in practice. Our model does not include stochasticity, although stochastic effects tend to hasten eradication. Furthermore, our model does not include age structure, which can influence prediction significantly, since sexual-mixing patterns tend to be highly age-dependent and heterogeneity will likely slow down transmission dynamics relative to homogenous mixing. The model also does not include type structure; this will have important consequences for replacement effects, ecological effects and other kinds of type interaction. Even eradicating types 16 and 18 may prove difficult within the context of high Canadian vaccine coverage; reintroductions from other populations with lower vaccine coverage, or vaccination heterogeneity among Canadian communities (eg rural or indigenous populations) may result in a recurring low epidemic. Such a situation currently exists with Hepatitis A, which has high vaccination coverage in Canada, but reintroductions occur from the United States [29]. Nevertheless, reducing the prevalence to the lowest levels possible, as predicted by our results, would obviously be the most desirable outcome.

A small proportion of adult vaccination can have a significant impact on eradication of targeted HPV types, when supplementing a large childhood vaccination program. This is due to the fact that childhood vaccination is a binary choice (children are either vaccinated or they are not), whereas adult vaccination is a rate, so that unvaccinated adults have a continual chance of being vaccinated, during their years in the sexually active cohort, if they are not infected first. However, there are drawbacks to adult vaccination: administering a vaccine to adults requires expensive physician office visits and compliance rates may be low. School-based vaccination is the cheapest and most efficient form of vaccination in the conventional sense of the term.

The mathematical model is a general one, with results that extend (via specific choice of parameters) to a variety of communities. We focus on Canada for specific implementation recommendations, but the model is applicable to other communities. It should also be noted that it may take decades, even centuries, to eradicate targeted types of the disease (Figure 4), but the most significant reduction should happen within 50 years. This is a remarkably fast timescale for disease eradication [30], but is possible because of the high efficacy and immunogenicity of the vaccine. However, even if eradication of targeted types is not achieved, vaccination of both children and adults is clearly desirable and efforts should still be made to vaccinate as widely as possible.

Future work will involve examining the differences between the Merck and GSK vaccines, the possibility of vaccines that wane and targeted vaccination for adults. A targeted vaccination program for adults could be implemented by offering free vaccination at STD clinics. This allows targeting of the subpopulation responsible for transmitting most sexually transmitted infections, although it should be noted that such a subpopulation is likely to already have high HPV incidence before attending the clinic. Such a model of targeted vaccination would require separate consideration for high-risk individuals. We will also generalise the model to include factors such as age-dependency in sexual partner rates, sexual activity outside of the vaccine age range among females and variable ages of sexual debut.

## Methods

### The model

The mathematical model that we have developed accounts for the vaccination of female children (*C*) and adult women (*A*). In particular, we assume that the pool of sexually active adults only includes women in an age range where they can still be vaccinated, as well as their male partners. Since men are not being vaccinated, we include them in our model only as age-equivalent cohort sexual partners of adult women. Thus, we account for the situation where older infected males may infect younger females who have or have not been vaccinated and who in turn infect other males. However, we assume that any men who interact with women in this cohort only do so for a finite amount of time (eg 10 years), although they may have multiple partners among this age group during that time. That is, while these men may have multiple partners among women aged 16–26, after a while they exhaust their contacts in this age group and are thus removed from the model.

We assume all vaccinated individuals are vaccinated before infection. Adult females vaccinated after infection are classified as "infected", since we assume that vaccination has no effect on this group. We consider vaccinated female children (*C*_*V*_) and female adults (*A*_*V*_) as those who received the vaccine and for whom the vaccine conferred immunogenicity. All other female individuals are functionally unvaccinated (*C*_*U*_, *A*_*U*_).

We treat men as either uninfected (*M*) or infected (*N*) with infection probability *β*_*M *_after interaction with an infected woman. Infection of an unvaccinated woman (*A*_*U*_) via contact with an infected man (*N*) occurs with transmission probability *β*_*N *_and results in an infected unvaccinated woman (*I*_*U*_). We assume that the vaccine confers no disease-modifying effects once infection has occurred; thus, we assume that the probability of infection is the same for men, regardless of whether their (infected) partners are vaccinated or unvaccinated. We also assume that the vaccine does not wane over time.

If the vaccine efficacy is not perfect, then vaccinated individuals have the potential to become infected. Infection of a vaccinated woman (*A*_*V*_) via contact with an infected man occurs with transmission probability *β*_*N*_, mediated by the efficacy (*ψ*) of the vaccine (*β*_*N *_(1 - *ψ*)), resulting in an infected vaccinated woman (*I*_*V*_). Thus, if the vaccine is 100% effective, transmission will be zero. Conversely, if the vaccine is completely ineffective, transmission is identical to the probability of infection for unvaccinated individuals, *β*_*N*_.

The rate of appearance of new females is *π*_*W *_while that of males is *π*_*M*_. The mortality rate of children is *μ*_*C *_and the leaving rate of adults (including mortality) is *μ*. In this model, we do not consider disease-induced mortality, since it does not play a role in removing individuals from the sexually active pool that we are considering [8,31]. Female children progress to adulthood at a rate *α*. Unvaccinated adult females who were not vaccinated as children may be vaccinated as adults via a function *f *denoting the rate at which unvaccinated adult females are vaccinated. This function depends on the proportion of adults vaccinated $\bar{p}$ and the immunogenicity of the vaccine in adults $\bar{\varepsilon }$:

$$f(\bar{\varepsilon }\bar{p})=\frac{c\bar{\varepsilon }\bar{p}}{1-\bar{\varepsilon }\bar{p}+\gamma },$$

with *c *an attenuation constant and *cγ*^-1 ^the maximum possible rate of vaccination, assuming perfect coverage and immunogenicity. The proportion of adult vaccinations is thus $\bar{\varepsilon }\bar{p}$. Since *f *corresponds to the rate of vaccination of adults (ie the rate at which unvaccinated adults leave the unvaccinated pool to go to the vaccinated pool), we want the rate to be zero if no adults are being vaccinated and we want the rate to be high if all adults are vaccinated (ie adults are vaccinated immediately upon entering). The model is illustrated in Figure 1.

### Mathematical analysis

Mathematically, our model is represented as follows:

$$\begin{array}{lll} \frac{dC_{U}}{dt} & = & \pi_{W}(1-\varepsilon p)-\alpha C_{U}-\mu_{C}C_{U}\\ \frac{dC_{V}}{dt} & = & \pi_{W}\varepsilon p-\alpha C_{V}-\mu_{C}C_{V}\\ \frac{dA_{U}}{dt} & = & \alpha C_{U}-f(\bar{\varepsilon }\bar{p})A_{U}-\mu A_{U}-\beta_{N}A_{U}N\\ \frac{dA_{V}}{dt} & = & \alpha C_{V}-f(\bar{\varepsilon }\bar{p})A_{U}-\mu A_{V}-(1-\psi )\beta_{N}A_{V}N\\ \frac{dI_{U}}{dt} & = & \beta_{N}A_{U}N-\mu I_{U}\\ \frac{dI_{V}}{dt} & = & (1-\psi )\beta_{N}A_{V}N-\mu I_{V}\\ \frac{dM}{dt} & = & \pi_{M}-\beta_{M}I_{U}M-\mu M-\beta_{M}I_{V}M\\ \frac{dN}{dt} & = & \beta_{M}I_{U}M-\mu N+\beta_{M}I_{V}M, \end{array}$$

with the function *f *given by (2).

The disease-free equilibrium satisfies

$$\begin{array}{lll} {\bar{C}}_{U} & = & \frac{\pi_{W}(1-\varepsilon p)}{\alpha +\mu_{C}}\\ {\bar{C}}_{V} & = & \frac{\pi_{W}\varepsilon p}{\alpha +\mu_{C}}\\ {\bar{A}}_{U} & = & \frac{\alpha C_{U}}{f+\mu }\\ {\bar{A}}_{V} & = & \frac{\alpha C_{V}+fA_{U}}{\mu }\\ \bar{M} & = & \frac{\pi_{M}}{\mu } \end{array}$$

and ${\bar{I}}_{U}={\bar{I}}_{V}=\bar{N}=0$. We assume $0\leq \varepsilon ,\bar{\varepsilon },p,\bar{p}\leq 1$. All other parameters are assumed to be positive. At the disease-free equilibrium, the Jacobian matrix is

$$J=\left[\begin{array}{cccccccc} -\mu_{C}-\alpha & 0 & 0 & 0 & 0 & 0 & 0 & 0\\ 0 & -\mu_{C}-\alpha & 0 & 0 & 0 & 0 & 0 & 0\\ \alpha & 0 & -f-\mu & 0 & 0 & 0 & 0 & -\beta_{N}A_{U}\\ 0 & \alpha & f & -\mu & 0 & 0 & 0 & -(1-\psi )\beta_{N}A_{V}\\ 0 & 0 & 0 & 0 & -\mu & 0 & 0 & \beta_{N}A_{U}\\ 0 & 0 & 0 & 0 & 0 & -\mu & 0 & (1-\psi )\beta_{N}A_{V}\\ 0 & 0 & 0 & 0 & -\beta_{M}M & -\beta_{M}M & -\mu & 0\\ 0 & 0 & 0 & 0 & \beta_{M}M & \beta_{M}M & 0 & -\mu \end{array}\right].$$

Then det (*J *- *λI*) = (- *μ*_*C *_- *α *- *λ*)^2 ^(- *μ *- *λ*)^2^(- *f *- *μ *- *λ*) det *M*, where

$$M=\left[\begin{array}{ccc} -\mu -\lambda & 0 & \beta_{N}A_{U}\\ 0 & -\mu -\lambda & (1-\psi )\beta_{N}A_{V}\\ \beta_{M}M & \beta_{M}M & -\mu -\lambda \end{array}\right].$$

Thus, the largest eigenvalue for *J *will be the largest eigenvalue for *M *and so we can reduce the problem to solving

*λ*^3 ^+ *αλ*^2 ^+ *βλ *+ *γ *= 0

where

*α *= 3*μ*

*β *= 3*μ*^2 ^- (1 - *ψ*)*β*_*M*_*β*_*N*_*MA*_*V *_+ *β*_*N*_*A*_*U*_*β*_*M*_*M*

*γ *= *μ*^3 ^- (1 - *ψ*) *μβ*_*M*_*β*_*N*_*MA*_*V *_+ *μβ*_*N*_*A*_*U*_*β*_*M*_*M*.

By the Routh-Hurwitz condition, all roots will have negative real parts if *α *> 0, *γ *> 0, and *αβ *- *γ *> 0. Clearly *α *> 0. We can write the third condition as

*αβ *- *γ *= 6*μ*^3 ^+ 2*γ*,

which will be positive if *γ *> 0. Thus, all roots will have negative real part if

*μ*^2 ^- *β*_*N*_*β*_*M*_*M*[*A*_*U *_+ (1 - *ψ*)*A*_*V*_] > 0.

Solving for *εp *in terms of $\bar{\varepsilon }\bar{p}$ and substituting equilibrium values, our eradication threshold is thus

$$\varepsilon p=\frac{1}{\psi \mu }\left[\mu +f(\bar{\varepsilon }\bar{p})(1-\psi )-\frac{\mu^{4}(\mu +f(\bar{\varepsilon }\bar{p}))(\alpha +\mu_{C})}{\beta_{M}\beta_{N}\pi_{M}\pi_{W}\alpha }\right],$$

with

$$f(\bar{\varepsilon }\bar{p})=\frac{c\bar{\varepsilon }\bar{p}}{1-\bar{\varepsilon }\bar{p}+\gamma }.$$

Differentiating (3), we have

$$\frac{\partial p}{\partial \bar{p}}=\frac{c\bar{\varepsilon }(1+\gamma )}{\varepsilon \psi \mu {\left(1-\bar{\varepsilon }\bar{p}+\gamma \right)}^{2}}\left[(1-\mu )\frac{\mu^{4}(\alpha +\mu_{C})}{\beta_{N}\beta_{M}\pi_{M}\pi_{W}\alpha }\right].$$

It follows that there is a critical vaccine efficacy ψ*, satisfying

$$\psi^{*}=1-\frac{\mu^{4}(\alpha +\mu_{C})}{\beta_{M}\beta_{N}\pi_{M}\pi_{W}\alpha }$$

such that if *ψ *<ψ*, then eradication of targeted types is not possible. Thus, even if the vaccine mounts an immune response 100% of the time and we can vaccinate 100% of the population, if the efficacy is below this threshold, then the disease will persist. See Figure 3A.

By setting *p *= 1 and $\bar{p}$ = 0, it also follows from (3) that there is a critical immunogenicity value ε*, satisfying

$$\varepsilon^{*}=\frac{1}{\psi }\left[1-\frac{\mu^{4}(\alpha +\mu_{C})}{\beta_{N}\beta_{M}\pi_{M}\pi_{W}\alpha }\right]$$

such that if *ε *<ε*, then even 100% childhood vaccination coverage will not eradicate targeted types of the disease.

From (3), when *p *= 1, we have

$$\varepsilon =\varepsilon^{*}+\frac{f(\bar{\varepsilon }\bar{p})}{\psi \mu }\left[(1-\psi )-\frac{\mu^{4}(\alpha +\mu_{C})}{\beta_{N}\beta_{M}\pi_{M}\pi_{W}\alpha }\right].$$

Define

$$\theta \equiv \frac{\psi \mu (\varepsilon^{*}-\varepsilon )}{\frac{\mu^{4}(\alpha +\mu_{C})}{\beta_{N}\beta_{M}\pi_{M}\pi_{W}\alpha }-(1-\psi )}.$$

For *ψ *> ψ*, and *ε *<ε*, *θ *> 0. It follows that the minimum level of adult vaccination required for eradication of targeted types, ${\bar{p}}^{*}$ satisfies

$${\bar{p}}^{*}=\frac{\theta (1+\gamma )}{\bar{\varepsilon }(c+\theta )}.$$

Since *θ *> 0, it follows that ${\bar{p}}^{*}$ > 0. If the immunogenicities *ε *and $\bar{\varepsilon }$ are not too small, then ${\bar{p}}^{*}$ < 1 (since *γ *is small). Note that we are not assuming that childhood immunogenicity is necessarily the same as adult immunogenicity. Thus, if childhood immunogenicity is below ε* (but not so small that the vaccine is nonfunctional), then there is a minimum level of adult vaccination coverage that must be achieved for eradication of targeted types. See Figure 3B.

To examine sensitivity of results on parameter variation, we used the output parameter as the proportion of adults who should be vaccinated in order to eradicate targeted types. Thus, rearranging equation (3), we have

$$\bar{p}=\frac{\delta (1+\gamma )}{\bar{\varepsilon }(c+\delta )},$$

where

$$\delta =\frac{\beta_{M}\beta_{N}\pi_{M}\pi_{W}\alpha }{\left(1-\psi )\beta_{M}\beta_{N}\pi_{M}\pi_{W}\alpha -\mu^{4}(\alpha +\mu_{C}\right)}\left(\varepsilon \mu \psi p+\frac{\mu^{5}(\alpha +\mu_{C})}{\beta_{M}\beta_{N}\pi_{M}\pi_{W}\alpha }-\mu \right).$$

## Competing interests

The author(s) declare that they have no competing interests.

## Authors contributions

ML performed the mathematical analysis and wrote the analysis section. RS conceived of the study, performed the numerical simulations and wrote the main part of the paper.

## Pre-publication history

The pre-publication history for this paper can be accessed here:


